# Surgical Approach in Metastatic Renal Cell Carcinoma: A Literature Review

**DOI:** 10.3390/cancers15061804

**Published:** 2023-03-16

**Authors:** Milena Matuszczak, Adam Kiljańczyk, Maciej Salagierski

**Affiliations:** Department of Urology, Collegium Medicum, University of Zielona Góra, 65-046 Zielona Góra, Poland

**Keywords:** metastases, kidney cancer, surgery

## Abstract

**Simple Summary:**

Methods of treatment should be carefully considered. Systemic therapy is recommended for patients susceptible to this method, as it will allow them to avoid surgery and postoperative complications. Surgery remains the standard of care for operable RCC resistant to systemic therapy.

**Abstract:**

The treatment of metastatic renal cell carcinoma has undergone considerable advances in the last two decades. Cytoreductive nephrectomy and metastasectomy retains a role in patients with a limited metastatic burden. The choice of optimal treatment regimen remains a matter of debate. The article summarises the current role of surgery in metastatic kidney cancer.

## 1. Introduction

**Figure 1:**
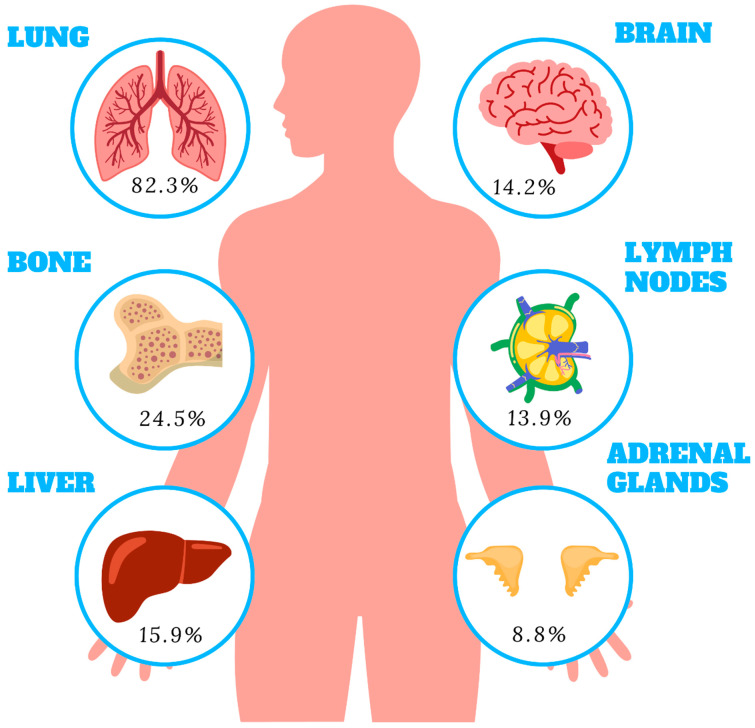
Most common sites of metastases in RCC.

Renal cell carcinoma (RCC) is the sixteenth most common cause of new cancers, with an estimated 431,288 new cases and 179,368 deaths for both genders in 2020 worldwide [1]. In the US, the estimated statistics for 2022 are 79,000 new cases and 13,920 deaths, respectively [2]. In the past, RCC used to be the most life-threatening cancer of the urinary tract [3] that typically manifested with a characteristic triad of symptoms: dull pain in the lumbar region, and a palpable mass in the abdomen and hematuria. Currently, these symptoms are encountered very rarely due to the fact that imaging examinations are more frequent than in previous years and the cancer is often diagnosed during follow-up examinations for another reason. Synchronous or metachronous metastases occur in about 20–30% of patients [4]. Five year OS for localized RCC exceeds 90% [5] compared to 20% for the metastatic type [6]. Surgery remains the standard of treatment for all stages [7]. Within it, we distinguish between complete (cMS) and incomplete (icMS) metastatectomy, and partial (PN) or complete (RN) nephrectomy. All types of surgical procedures were included, i.e., laparoscopic, robotic or classic forms. Approximately one-third of RCC manifests with metastases, the most common sites of which are the lung, bone, liver, lymph nodes, adrenal gland, and brain (Figure 1) [8]. Histologically, a distinction is made between four grades on the Fuhrman Nuclear Grade scale, which takes into account the shape and size of the cell nucleus of RCC [9]. Patients at an advanced stage with metastases or recurrence (stage IV) are treated with surgery and/or systemic therapy according to the latest NCCN guidelines [10]. Clinicopathological factors affect both the course of surgery and patient survival, and should be taken into account when selecting a therapy and evaluating its effectiveness.

Our work focuses on the relevance of surgery in the treatment of metastatic RCC, as well as the criteria that should be considered to select patients who will benefit most from such therapy.

## 2. Methods

This literature review was conducted using a database search of PubMed/MEDLINE. Inclusion criteria for this review included:

(a) articles published after 2016;
(b) based on a screening of the references of the included articles, papers published before 2016 and relevant to the topic of this review were also included (25 articles);
(c) articles including keywords such as: RCC, surgery, metastasis, vena cava invasion, mRCC, nephrectomy, metastatectomy, cytoreductive nephrectomy, partial nephrectomy, RARN, renal surgery using search term database = specific-medical subject headings terms in various combinations appropriate to the purpose of the study.

Criteria for exclusion from the literature review included:

(a) articles on animals, tissues, cell lines;
(b) involving more than one cancer, e.g., semi-occurrence of kidney and prostate or bladder cancer;
(c) papers focusing on the technical feasibility and specification of measurement methods rather than method and clinical utility;
(d) publications based on small cohorts (less than 5 patients).

All discrepancies in data extraction were resolved by consensus with the co-authors.

## 3. Results

Table 1 presents results obtained by different research groups for various therapeutic approaches for mRCC. The table excludes review papers and includes three papers that were published before 2016. The results of articles featuring treatment of a single type of metastasis are not included in the table.

### 3.1. Nephrectomy

**Figure 2:**
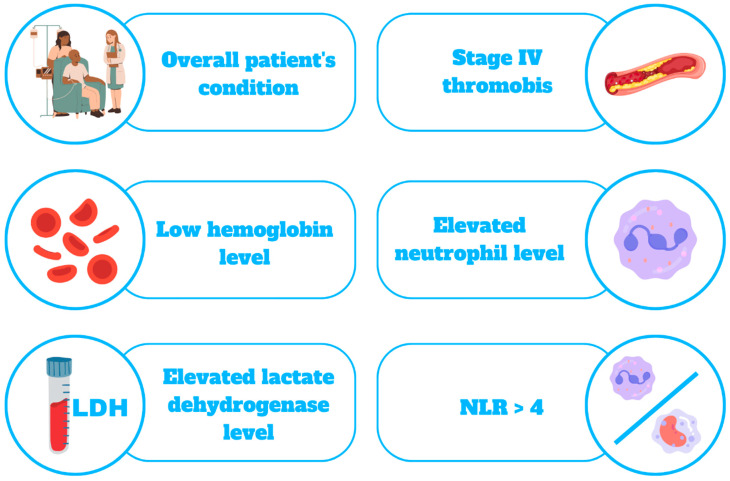
Selected prognostic factors of RCC. NLR—neutrophil to lymphocyte ratio, LDH—lactate dehydrogenase.

RN is the treatment of choice for advanced metastatic and localized RCC. Patient selection is based on the stage of the disease and the presence of prognostic factors (Figure 2) in patients with mRCC [23].

Trinh et al. studied 16,285 patients who underwent cytoreductive nephrectomy (CN) for mRCC, 31% of whom had one or more complications [24]. Studies have demonstrated that this method achieves optimal local control (LC), eliminates the source of metastasis, inhibits the potential further development of metastasis, and removes the active source of immunosuppression. In addition, RN allows half of the patients to prolong survival and delay the delivery of systemic treatment. The addition of nephrectomy to interferon therapy allowed for the achievement of not only improved survival (percentage), but also longer survival times [23]. In addition to immunotherapy, the use of CN in targeted therapy (TT) also provided benefits—reducing the toxicity profile, and prolonging PFS and OS [25,26]. Also worthy of mention is the CARMENA study, which reported similar results with sunitib treatment compared to sunitib preceded by surgery. Unfortunately, the trial did not meet the target enrollment of 576 patients and was terminated prematurely [27]. In a randomized trial called SURTIME, researchers reported better OS in a group of patients with deferred CN preceded by three cycles of sunitinib compared to primary CN followed by sunitinib therapy, therefore they suggest administering sunitib before CN to identify patients refractory to systemic therapy before implementing surgical approaches [28].

In addition, RN is applicable in the treatment of IVC. It is performed at stage I and II of IVC invasion, while stage III and IV require additional methods to achieve a bloodless surgical field, and these include cardiopulmonary bypass (CPB) and deep hypothermic circulatory arrest (DHCA) [29]. The use of these methods, while reducing the risk of perioperative death, carries a significant risk of perioperative complications [30].

According to NCCN recommendations, treatment with CN prior to systemic therapy is the recommended method in selected patients with a possibly surgically operable primary tumor. The study authors also indicate that patients with good general condition, prognostic factors and lung-only metastases undergoing CN before systemic therapy had the best results. For patients with unresectable tumors, the NCCN recommends conducting a tissue biopsy for histopathological confirmation of RCC, which allows the treatment to be tailored [10].

The AUA recommends performing adrenalectomy while undergoing surgical resection of a renal tumor if imaging or intraoperative findings indicate adrenal metastasis [31,32].

Nephrectomy is also used in palliative patients [33] for unmanageable hemorrhage and when paraneoplastic symptoms appear, but this procedure is rare [23].

### 3.2. IVC Thrombus

In 4–10% of patients, RCC infiltrates the of inferior vena cava (IVC), and 5–15% of them develop thrombus formation. Patients in this group have a higher risk of cancer recurrence, even after successful thrombus removal. We distinguish between four levels (Table 2) of thrombus invasion [34]. Extremely rare (1%) but carrying a very high risk is clot invasion into the right ventricle (level 4) [3].

Adequate patient selection seems to be important, for example, laparoscopy is used for IVC I, RAPN for IVC II, and open surgery for stage T3b/T3c/T4 and IVC III, IV (using CPB and DHCA in addition). Unsurprisingly, mortality in the group using CPB was much more frequent than in the other cohorts. RAPN had a higher incidence of postoperative complications compared to the other two cohorts. Laparoscopy usually qualifies patients who tend to be ‘healthier’ (with fewer metastases, lower BMI and younger, and a smaller thrombus diameter), which implies better outcomes.

In a cohort with RCC and level I-II thrombus treated by laparoscopy, those after transperitoneal radical nephrectomy had satisfactory outcomes and none required conversion to open surgery [35]. Moreover, the possibility of performing this operation laparoscopically has also been reported by other clinicians [36]. They undertook surgery not only with a thrombus level II but also with a level IV and achieved good therapeutic results in this group as well, thus demonstrating that this approach could be an alternative to open surgery that is safe and technically feasible.

Robotic assisted radical nephrectomy with tumour thrombus (RA-RNTT) patients have a longer duration of surgery but less intraoperative and postoperative blood loss and number of blood transfusions and a shorter period of hospitalisation than open radical nephrectomy with tumour thrombus (O-RNTT) surgery [12]. This provides evidence supporting the benefit of this method in any patient group.

IVC placement affects OS, as evidenced in the article by Lenis et al. [17] For renal vein thrombus, OS = 24.0 vs. 9.2 months for CN vs. non-CN, respectively. For infradiaphragmic thrombus, OS = 22.3 vs. 11.5 months for CN vs. non-CN, respectively. For supradiaphragmic thrombus, OS = 13.1 vs. 10.3 months for CN vs. non-CN, respectively. The researchers concluded that the benefits of CN are limited to thromboses located below the diaphragm.

### 3.3. Metastectomy (MS)

Metastatectomy is a common procedure used in advanced cancer and consists of surgical removal of metastases secondary to the primary tumour from other organs. The most commonly treated metastases are those to the lungs, brain, adrenal glands, liver and pancreas [37]. There are many retrospective studies evaluating the role of metastatectomy in the treatment of mRCC, but there are no randomized trials [38].

According to EAU guidelines, metastases are potentially curable only if all tumor sites are removed. This applies to patients with a primary localized tumor, or with single or oligo metastases. CN is a palliative treatment for which systemic therapy is required [39].

Patients targeted for metastatectomy and nephrectomy according to NCCN guidelines are those with primary RCC and oligometastases or those who have developed oligometastases after a long disease-free interval (DFI) from nephrectomy. Metastases that are prone to this procedure are the bones, brain and lungs. The primary tumor and metastases can be removed during the same procedure or at different times.

#### 3.3.1. Lung Metastases

The International Registry of Patients with Lung Metastases, based on the three parameters of resectability, disease-free interval DFI and number of metastases, has created a scale (Table 3) that classifies patients into four prognostic groups [40].

Later, the Munich scale was also developed [41] based on prognostic factors (Table 4).

Lung metastases are associated with the best prognosis with relatively low mortality and morbidity. Ouzaid et al. [42], in a systematic review, derived a 1-, 3-, 5-, and 10-year OS of 84%, 59%, 43% and 20%, respectively. The authors reported that [43] lung metastases have been shown to have higher OS after MS compared to TT and immunotherapy.

Procházková et al. studied a group of 35 mRCC patients undergoing pulmonary metastasectomy (PM) and reported a 3-year OS and PFS at 63.5% and 39.4%, respectively, and a 5-year OS and PFS at 44.9% and 29%, respectively. They also concluded that OS and PFS were significantly dependent on the size of the metastasis, with corresponding hazard ratios of 1.38 and 1.41 for each 10 mm in size of the largest metastatic site [44]. In an article analyzing 27 mRCC patients undergoing PM, researchers demonstrated that metastasis size ≥2 cm and DFI ≥5 years are independent prognostic factors for survival. They also stated that metastasis size ≥2 cm was the only independent prognostic factor that affected DFI [45].

#### 3.3.2. Adrenal Metastases

Adrenal metastasis can be both synchronous and metachronous to renal cancer. We distinguish between unilateral, contralateral, and bilateral, and it can also occur as disseminated disease or a single metastasis.

Importantly, patients with isolated adrenal metastasis have significantly longer survival compared to those who also have metastasis to other organs. It was also shown that there was no significant difference between single and multiple adrenal metastases, and that surgical removal of the metastasis provided a successful cure. In addition, no recurrence was observed during the 82.9-month follow-up period [46].

#### 3.3.3. Liver Metastases

Metastases occurring in the liver are associated with a poor prognosis and are often co-related to the presence of disseminated cancer. They can occur as synchronous or metachronous disease and as a single liver metastasis. Unfortunately, liver metastases are associated with frequent recurrence, which occurs in about half of patients (41.9%), is both intra- and extrahepatic, and significantly affects overall survival [47].

Of note, significantly better survival was shown for patients who had MS surgery compared to those who did not (142 vs. 27 months) [48]. The complication risk ranges from 13–23%. The 5-year OS ranges from 38–62%, with the completeness of resection being a significant factor affecting OS. Moreover, OS is affected by other features: the grade of the primary tumour, the disease-free interval, Eastern Cooperative Oncology Group (ECOG) status, and the presence of metastases outside the liver [47].

#### 3.3.4. Pancreatic Metastases

RCC metastases to the pancreas are rare, accounting for 2–5% of all metastases [49]. Pancreatic metastases are associated with a good prognosis, grow slowly, and have a long DFI (>10 years) [49]; thus, metastases to the pancreas are often detected accidentally [50]. However, the surgical procedure is challenging to perform and is associated with a fairly high mortality rate (they have a similar prognosis to lung metastases, but the procedure itself is much more difficult to conduct). Different types of resection can be used: pancreaticoduodenectomy, distal pancreatectomy, enucleation, and enucleoresection [51]. Similarly to the liver, performing an MS was associated with significantly better OS; 5-year OS was achieved in 88% of patients after the intervention vs. 47% for patients without it [52]. Fikatas et al. demonstrated a 5-year OS = 71.4% and indicated that 22.2% of patients developed complications after pancreatic surgery. Moreover, in their opinion, this is one of the best therapeutic options for this patient population, only better results can be achieved when it is combined with TT [53].

#### 3.3.5. Other Metastases

Central nervous system metastases were associated with the worst prognosis, as patients in this group had a 5-year survival rate of only 18% [23]. A study [54] on a population of 1780 patients with clinically positive lymph nodes showed that their excision improved OS by 3% for each node excised; however, this group of patients is at lower risk of survival due to other comorbid factors.

## 4. Discussion

By 2005, reports already showed the benefit of total MS, which had achieved a two-fold lower risk of death [55]. More recent work shows that currently cMS achieves as much as a three-fold reduced mortality risk. Palumbo et al. [8] revealed that MS was associated with lower overall mortality (OM) and a 2-month longer median OS.

The meta-analysis [31] proved a significant improvement in CSS and OS with cMS versus cohorts with incomplete or no MS (median 121.9 mts for MS vs. 28.1 mts for nonMS vs. 81.5 mts for icMS). In addition, another meta-analysis [32] based on the data of more than 2200 patients with metastatic RCC found that MS allowed patients to survive 36.5–142 months, which was much longer than the cohort that did not undergo this procedure (8.4–27 months). A study of the Japanese population showed similar results in terms of improved survival, with 44.3 vs. 16.4 months for the MS and no procedure cohorts, respectively [33]. A systematic review of 56 studies demonstrates the benefit of MS prior to systemic therapy—significant improvement in OS [16]. In addition, cohorts that underwent total MS were less likely to require systemic therapy—as many as 46.7% of patients required no further interventions. This is undoubtedly an asset to improve the quality of life of oncology patients [37]. A comparative analysis [21] was performed on a population of 325 patients who were divided into three cohorts: cMS, icMS and non-MS. The following 2- and 4-year OS were achieved: 81.82% and 51.52% for cMS; 51.72%, 13.79% for icMS; and 22.05% and 4.94% for non-MS. An improved 5-year survival from 30% to 45% was achieved in the MS group [56]. With complete surgical resection, a significant improvement in 5-year CSS (49.4%) and median CSS by 4.8 years (compared to the group that underwent icMS) was achieved.

The CARMENA and SURTIME trials indicate the efficacy of treatment with sunitinib alone, and delayed CN after three cycles of sunitinib. The authors stress the importance of appropriately classifying patients for treatment. The use of systemic therapy first may help to identify patients resistant to systemic therapy. Thus, extensive treatments with possible complications will be reserved for patients in whom safer methods are not effective [27,28].

Nephrectomy in combination with MS allows the patient to be ‘regressed’ to a disease-free state, and the best effect is achieved when it is combined with neoadjuvant therapy. Patients with a benign disease course may benefit significantly from the surgery [23]. This approach, called aggressive surgery, allowed patients to have a 3- and 5-year OS of 99% and 31%, respectively. Studies indicate that both MS and CN lead to better outcomes (improved OS and low tumour burden (LTB) when performed prior to systemic treatment with TT or Immune Checkpoint Inhibitors (ICI). [57]. In addition, the combination of CN + anti-VEGF and mTOR results in a significant improvement in mean OS compared to surgery alone [13]. Li et al. [58] showed that in the mRCC patient population, prolonged OS was achieved with total resection and TT. The use of MS in combination with systemic therapy has the additional advantage of a drug holiday, which prevents the side effects of therapy [58]. Although methods of systemic therapy are constantly improving, surgery still plays a significant role, and a combination of both methods gives the best therapeutic effect.

Several articles show the benefit of using CN (Table 1). Hanna et al. [13], based on a cohort of 15390 patients, reports 2-year OS = 39.1% vs. 17.1% for CN vs. non-CN. Graham et al. [14] reports (for *n* = 351) a median OS = 16.3 vs. 8.6 months for CN vs. non-CN. Mathieu et al. [15] reports (for n = 351) a median OS = 38.1 vs. 16.4 months for CN vs. non-CN. Another article [16] analyzing 1113 patients showed that for CN, 1-year OS and CSM were 70.7% and 27.9%, respectively. Compared to no surgery, the 1-year OS and CSM were 43.6% and 60.3%, respectively. This indicates the advantage of CN over no intervention.

DiNatale et al. [19] showed that a centimeter difference in primary tumor size is associated with a 10–31% change in the risk of death. They also showed that the size of the primary tumor affects the number of metastases. Solid organs and core metastases have a better prognosis due to ease of surgical removal, and result in higher CSS (84% and 69%, respectively) [59,60]. A worse prognosis, however, is associated with the presence of metastases in the central nervous system or liver [23].

Yuan et al. [3] analysed the data and showed that open surgery has a longer operation time, hospitalisation, and higher blood loss compared to laparoscopic and robotic methods. In the coming years, we can expect to see a higher proportion of laparoscopic and robotic surgery in the future for both RN [3] and MS [57].

The success of the surgical procedure and the prognosis of the patient are determined by prognostic factors (Figure 2). Local tumor spread is more prognostically significant than the degree of IVC infiltration (Table 2). The patient’s general condition, low hemoglobin, elevated lactate dehydrogenase, grade IV thrombosis, retroperitoneal adenopathy, elevated relative neutrophil count, and neutrophil to lymphocyte ratio (NLR) above 4 are independent unfavorable predictive factors (Figure 2) [11]. In addition, hypercalcemia and weight loss greater than 10%, in addition to elevated erythrocyte sedimentation rate are recognized prognostic factors [23]. Distant metastasis is a significant risk factor for postoperative mortality. Higher mortality is also associated with sarcomatoid features and lymph node metastasis.

The risk of selection bias in our study is related to the inclusion in the review of papers of different types, including non-randomized papers, with different study durations, group sizes, and considering heterogeneous patients (different ages, backgrounds, and different stages of cancer). In addition, the publications used various equipment, both for the analysis of the samples taken and during the therapeutic process. Moreover, the same version of software was not used, which does not allow for the direct comparison of results.

## 5. Conclusions

Every group of patients with mRCC can benefit from surgical treatment as long as their overall condition allows them to undergo this procedure. Palliative patients may also benefit, but they are more likely to respond to systemic treatment, while younger patients in good general condition with favourable prognostic factors are most likely to succeed after CN [23].

It seems that for patients with mRCC, MS should be an integral part of the treatment, because, in each population, there have been benefits from this therapeutic modality. Therefore, whenever the patient’s general condition allows for surgery, it should be considered [56]. The optimal therapy should be tailored individually and a surgical approach should always be considered.

## Figures and Tables

**Table 1:** Comparison of results for surgical management of mRCC.

Study	Study Group	Study Goal	Results		
Palumbo et al. [8]	*n* = 3654	MS vs. no MS comparison	MS (*n* = 437) Median OS = 11 months	No MS (*n* = 3217) Median OS = 9 months	
Peyton et al. [11]	*n* = 447	Utility of NLR in mRCC with tumor thrombus undergoing CN	Median follow-up = 24 months Mean OS = 50.6 months		
Rose et al. [12]	*n* = 75	O-RNTT vs. RA-RNTT (level I, II) comparison	O-RNTT (*n* = 27) Median follow-up = 79 months Mean OS = 48.7 months	RA-RNTT (*n* = 24) Median follow-up = 24 months Mean OS = 50.6 months	
Hanna et al. [13]	*n* = 15,390	CN vs. non-CN survival analysis	CN (*n* = 5374) 1 yr OS = 62.7% 2 yr OS = 39.1% 3 yr OS = 27.7%	Non-CN (*n* = 10,016) 1 yr OS = 34.7% 2 yr OS = 17.1% 3 yr OS = 9.8%	
Graham et al. [14]	*n* = 353	CN vs. non-CN survival analysis	CN (*n* = 109) Median OS = 16.3 months PFS = 5.1 months	Non-CN (*n* = 244) Median OS = 8.6 months PFS = 3.4 months	
Mathieu et al. [15]	*n* = 351	CN vs. non-CN survival analysis	CN (*n* = 298) Median OS = 38.1 months	Non-CN (*n* = 53) Median OS = 16.4 months	
Zhao et al. [16]	*n* = 1113	CN vs. no surgery comparison	CN (*n* = 618) Median OS = 26 months 1 yr OS = 70.7% 1 yr CSM = 27.9%	No surgery (*n* = 495) Median OS = 9 months 1 yr OS = 43.6% 1 yr CSM = 60.3%	
Lenis et al. [17]	*n* = 4962	CN vs. non-CN survival analysis	CN (*n* = 2460) 1 yr OS = 66.65% 2 yr OS = 39.16% 5 yr OS = 2.25%	Non-CN (*n* = 1502) 1 yr OS = 40.75% 2 yr OS = 19.04% 5 yr OS = 0.4%	
	*n* = 2056	CN vs. non-CN survival analysis (with IVC thrombus)	Renal vein thrombus CN median OS = 24.0 months Non-CN median OS = 9.2 months	Infradiaphragmic thrombus CN median OS = 22.3 months Non-CN median OS = 11.5 months	Supradiaphragmic thrombus CN median OS = 13.1 months Non-CN median OS = 10.3 months
Lenis et al. [18]	*n* = 1047	PN vs. RN comparison	PN (*n* = 381) 1 yr OS = 76% 2 yr OS = 49.61% 5 yr OS = 13.12% Median follow-up = 23.7 months	RN (*n* = 666) 1 yr OS = 64.7% 2 yr OS = 42.04% 5 yr OS = 9.46% Median follow-up = 17.4 months	
DiNatale et al. [19]	*n* = 1082	Assessing tumor size on survival of PN patients	MSK cohort (*n* = 304) Median follow-up = 44.2 months 2 yr OS = 65.8% 5 yr OS = 31.2%	IMDC cohort (*n* = 778) Median follow-up = 28.7 months 2 yr OS = 65.3% 5 yr OS = 28.3%	
Nini et al. [20]	*n* = 46	Comparison of OS in cM0 vs. cM1 RN with ECC and DHCA cohort	cM1 (*n* = 15) 1 yr OS = 46% 2 yr OS = 23% 3 yr OS = 23%	cM0 (*n* = 31) 1 yr OS = 89% 2 yr OS = 75% 3 yr OS = 63%	
You et al. [21]	*n* = 325	cMS vs. icMS vs. no-MS comparison	cMS (*n* = 33) 2 yr OS = 81.82% 4 yr OS = 51.52%	icMS (*n* = 29) 2 yr OS = 51.72% 4 yr OS = 13.79%	No-MS (*n* = 263) 2 yr OS = 22.05% 4 yr OS = 4.94%
Tornberg et al. [22]	*n* = 97	cMS vs. icMS comparison	cMS (*n* = 46) 5 yr OS = 59% 1 yr RFS = 29.79% 5 yr RFS = 4.26%	icMS (*n* = 51) 5 yr OS = 45%	

cMS—complete metastectomy, CN—Cytoreductive nephrectomy, DHCA—Deep hypothermic circulatory arrest, ECC—Extracorporeal circulation, icMS—Incomplete metastectomy, IMDC—International Metastatic Database Consortium, MS—Metastectomy, MSK—Memorial Sloan Kettering, mRCC—Metastatic renal cell carcinoma, NLR—Neutrophil to Lymphocyte Ratio, OS—Overall survival, O-RNTT—Open radical nephrectomy with tumor thrombus, RA-RNTT—Robot assisted radical nephrectomy with tumor thrombus, RCC—Renal cell carcinoma.

**Table 2:** IVC tumor thrombus level in RCC.

Tumor Thrombus Level	Clinical Description
I	Extension to the renal vein only
II	Infrahepatic IVC extension
III	Intrahepatic IVC extension
IIIa	Intrahepatic IVC extension (above the liver edge, below the level of hepatic veins)
IIIb	Hepatic IVC extension
IIIc	Suprahepatic, infradiaphragmatic extension
IIId	Suprahepatic, supradiaphragmatic, infraatrial extension
IV	Atrial extension

**Table 3:** International Registry of Lung Metastases score.

Parameter	Prognostic Group			
	Group 1	Group 2	Group 3	Group 4
Resectability	resectable	resectable	resectable	unresectable
Risk factors:	0	1	2	N/A
Disease-free interval (DFI)	>36 mo	<36 mo	<36	N/A
Number of metastases	and	or	and	N/A
	single	multiple	multiple	N/A

N/A—not applied.

**Table 4:** Munich score system for lung cancer.

Prognostic Factors	Groups
Pleural infiltration	I—low risk: no risk factors, R0
Synchronous manifestation of primary RCC and pulmonary metastases	II—intermediate risk: ≥1 risk factor, R0
Nodal status of the primary tumor	III—high risk: R1 or R2
Metastasis size > 3 cm	
Mediastinal and/or hilar lymph node metastases	
Completeness of metastatectomy	

## Data Availability

Not applicable.

## Abbreviations

cMS	Complete metastatectomy
CN	Cytoreductive nephrectomy
CPB	Cardiopulmonary bypass
CSM	Cancer specific mortality
CSS	Cancer specific survival
DFI	Disease free interval
DHCA	Deep hypothermic circulatory arrest
ECC	Extracorporeal circulation
ECOG	Eastern cooperative oncology group
ICI	Immune checkpoint inhibitors
icMS	Incomplete metastatectomy
IMDC	International Metastatic Database Consortium
IVC	Inferior vena cava
LC	Local control
LTB	Low tumor burden
mRCC	Metastatic renal cell carcinoma
MS	Metastatectomy
MSK	Memorial Sloan Kettering
mTOR	Mammalian target of rapamycin
NLR	Neutrocyte to lymphocyte ratio
NSS	Nephron sparing surgery
OM	Overall mortality
O-RNTT	Open radical nephrectomy with tumor thrombus
OS	Overall survival
PM	Pulmonary metastasectomy
PN	Partial nephrectomy
RAPN	Robot assisted partian nephrectomy
RA-RNTT	Robot assisted radical nephrectomy with tumor thrombus
RCC	Renal cell carcinoma
RN	Complete nephrectomy
TT	Targeted therapy
VEGF	Vascular endothelial growth factor
